# Pediatric *Tuina* for functional constipation in children: study protocol for a randomized controlled trail

**DOI:** 10.1186/s13063-022-06678-y

**Published:** 2022-09-05

**Authors:** Xinghe Zhang, Luan Hu, Li Li, Yuanwang Wang, Can Zhang, Jinyan Su, Hua Di, Qing Gao, Xiantao Tai, Taipin Guo

**Affiliations:** 1 School of Second Clinical Medicine, Yunnan University of Chinese Medicine, Kunming, China; 2 Department of Tuina, Second Affiliated Hospital of Yunnan University of Chinese Medicine, Kunming, China; 3 Department of Tuina, Yunnan Province Hospital of Traditional Chinese Medicine, Kunming, China; 4 Department of Science and Technology, Yunnan University of Chinese Medicine, Kunming, China; 5 Department of Chinese Medicine, Kunming Children’s Hospital, Kunming, China; 6 Department of Acupuncture and Tuina, Zhejiang Provincial People’s Hospital, Hangzhou, China; 7 Department of Pediatric Tuina, Shandong Province Hospital of Traditional Chinese Medicine, Jinan, China

**Keywords:** Pediatric *Tuina*, Functional constipation, Randomized controlled trial

## Abstract

**Background:**

Functional constipation (FC) is a common functional gastrointestinal disorder, which brings many negative impacts to the children’s daily life. Pediatric *Tuina* has been proved to be a potential therapy for FC. However, the evidence for its effectiveness and safety is insufficient due to the lack of high-quality study. This study aims to evaluate the efficacy and safety of pediatric *Tuina* for children with FC.

**Methods/design:**

This study is a randomized, controlled, multicentre, clinical trial. We will include 176 children with FC from five hospitals. The participants will be randomly allocated into two groups: the pediatric *Tuina* group and the Medilac-Vita group. This study will include a 1-week actual treatment period and a 2-week follow-up period. Primary outcomes are weekly spontaneous bowel movements and weekly complete spontaneous bowel movements. The secondary outcomes are effective rate, stool form, distress sensation, and glycerine enema rate. The assessment will be performed each week. Adverse event will be monitored in the treatment period and follow-up period.

**Discussion:**

This study is designed to evaluate the efficacy and safety of pediatric *Tuina* for children with FC, and we hypothesize that pediatric *Tuina* is more effective than probiotics. It will provide reliable evidence and support for the treatment of FC by pediatric *Tuina*.

**Trial registration:**

This protocol was registered in the Chinese Clinical Trial Registry (ChiCTR2100046485).

**Supplementary Information:**

The online version contains supplementary material available at 10.1186/s13063-022-06678-y.

## Background

Functional constipation (FC), a common functional gastrointestinal disorder (FGID), which is characterized by infrequent, difficult and incomplete defection [1, 2]. According to the survey report, the prevalence of FC in children ranges from 0.7% to 29.6% (median 12%) [3–5]. Although FC is not life-threatening, it significantly influences the patients’ quality of life and consumes many medical resources [6–8]. Currently, there are two broad categories of treatments for FC: pharmacological and nonpharmacological [9]. Pharmacological treatments have limited therapeutic effects but considerable side effects, while the symptom tends to recur [10–12]. Nonpharmacological treatments, such as physical activity, fiber, and fluid intake, are recommended to regulate bowel movement, whereas the evidence is still insufficient and the strength of recommendation is weak [9, 13, 14]. Therefore, it is urgent to find an effective and safe therapy for FC in children.

Pediatric *Tuina* is a special massage therapy based on traditional Chinese medicine (TCM), which can be traced back to Qin Dynasty (220 B.C.) and the complete system was established in the Ming Dynasty (1601 A.D.) [15]. Up to now, many universities of TCM offer the subjects of pediatric *Tuina* for medicos [16]. The special meridian-acupoint theory is different from adults, most acupoints are located on the finger, palm, and forearm, while few acupoints are located on the head, abdomen, and back [17]. The manipulations such as rubbing, pushing, pinching, kneading, and vibrating are characterized by light, fast, and gentle [18].

In recent years, many randomized controlled trials (RCTs) have indicated that pediatric *Tuina* may be an effective treatment for FC in children [19]. While in other countries, different massage manipulations have also been used for constipation, such as abdominal massage, reflexology, Dalk (Iranian traditional massage), etc. [20–22]. However, the inadequacy of the study design restricts the quality and credibility [19, 22–24]. Well-designed high-quality RCTs are urgently needed.

### Objective

This randomized controlled trial aims to (a) evaluate the efficacy of pediatric *Tuina* for children with FC, (b) evaluate the safety of pediatric *Tuina* for children with FC, and (c) compare the effect of pediatric *Tuina* with Medilac-Vita.

## Methods/design

### Study design

**Fig. 1:**
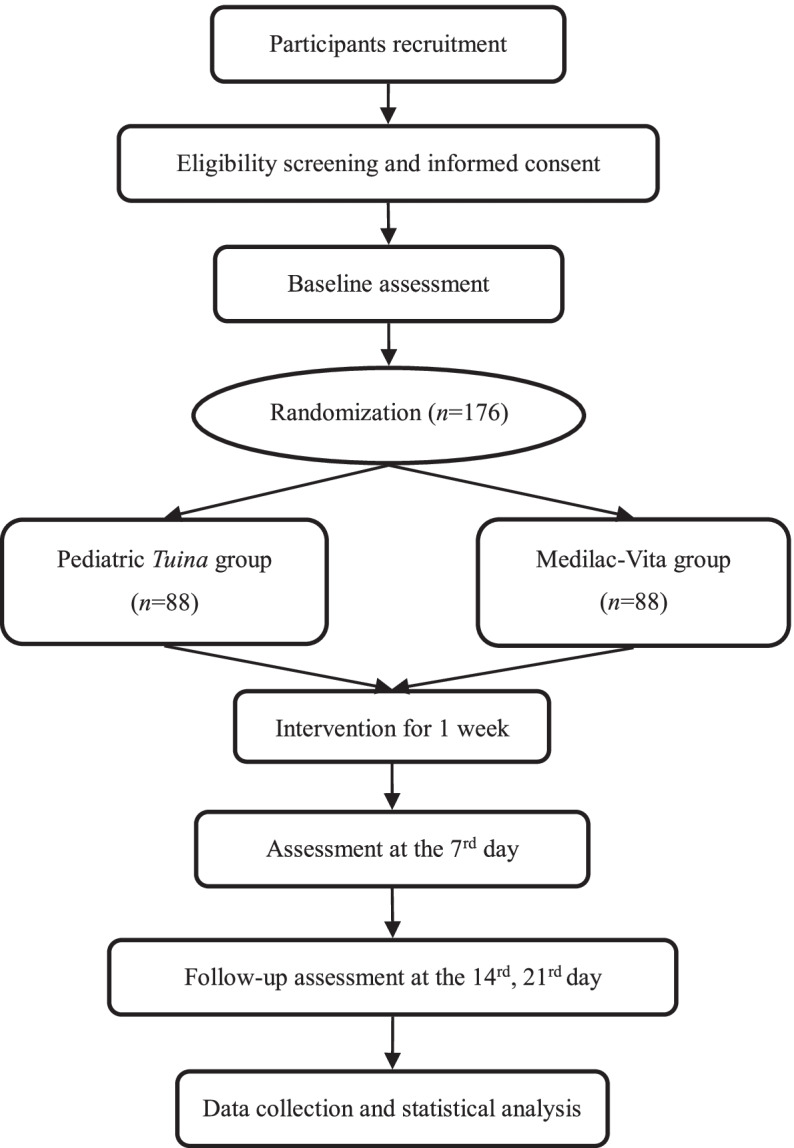
Flowchart of study design

This study is a prospective, randomized, multicentre, controlled design, and it will be conducted from December 2021 to December 2024. We will recruit 176 participants from 5 hospitals in 3 different provinces (Yunnan province, Zhejiang province, and Shandong Province). The eligible participants will be randomly allocated to 2 groups in a 1:1 ratio. The randomization sequence will be computer generated by the Clinical Research Center of the Yunnan University of Chinese Medicine. Every participant will receive a 1-week treatment period and a 2-week follow-up. Basic information and outcomes will be assessed at baseline and after each week. The flowchart is shown in Fig. 1, and the schedule is shown in Table 1.

**Table 1:** Schedule of the study

Item	Days													
	−7	–	0	1	2	3	4	5	6	7	–	14	–	21
**Eligibility screen**	×													
**Informed consent**	×													
**Baseline**	×	×	×											
**Allocation**			×											
**Pediatric** ***Tuina***			×		×		×		×					
**Medilac-Vita**			×	×	×	×	×	×	×					
**Follow-up**										×	×	×	×	×
**Stool dairy**	×	×	×	×	×	×	×	×	×	×	×	×	×	×
**Assessment**														
**SBMs**			×							×		×		×
**CSBMs**			×							×		×		×
**Effective rate**			×							×		×		×
**m-BSFS**			×							×		×		×
**Distress sensation**			×							×		×		×
**Glycerine enema rate**			×							×		×		×
**Participants safety**														
**Adverse events**			×	×	×	×	×	×	×	×	×	×	×	×

This trial is reported in line with the Standard Protocol Items Recommendations for Interventional Trials (SPIRIT) guidelines, and details are provided in Additional file 1. This trial follows the principles of the Declaration of Helsinki (Version Edinburgh 2000). This protocol was registered in the Chinese Clinical Trial Registry (ChiCTR2100046485) and approved by the Hospital Ethics Committee of Second Affiliated Hospital of Yunnan University of Chinese Medicine (2021–014).

### Participants

This study will include 176 participants from the departments of Acupuncture and *Tuina* in Second Affiliated Hospital of the Yunnan University of Chinese Medicine, Yunnan Province Hospital of TCM, Kunming Children’s Hospital, Zhejiang Provincial People’s Hospital, and Shandong Province Hospital of TCM. The participants will be appropriately compensated after they complete the entire procedure. However, parents or legal guardians of the participants have the right to withdraw from this study at any time, for any reason. And they will not face any discrimination or hardship in the hospital. The participants will be children with FC and they must meet the following eligibility criteria.

### Inclusion criteria

1. Children aged 1–7 years.
2. Children who meet the diagnostic criteria for functional constipation (Rome IV) [25, 26]. Must include 1 month of at least 2 of the following in infants up to 4 years of age: (1) 2 or fewer defecations per week; (2) history of excessive stool retention; (3) history of painful or hard bowel movements; (4) history of large-diameter stools; and (5) presence of a large fecal mass in the rectum. In toilet-trained children, the following additional criteria may be used: (6) at least 1 episode/week of incontinence after the acquisition of toileting skills and (7) history of large-diameter stools that may obstruct the toilet. For children over 4 years of age, there must include 2 or more of the following occurring at least once per week for a minimum of 1 month with insufficient criteria for a diagnosis of irritable bowel syndrome: (1) 2 or fewer defecations in the toilet per week in a child of a developmental age of at least 4 years; (2) at least 1 episode of fecal incontinence per week; (3) history of retentive posturing or excessive volitional stool retention; (4) history of painful or hard bowel movements; (5) presence of a large fecal mass in the rectum; and (6) history of large diameter stools that can obstruct the toilet.
3. Parents or legal guardians are motivated to participate in the trial.
4. Parents or legal guardians sign written informed consent.

### Exclusion criteria

1. A history of intestinal surgery.
2. Receiving gastrointestinal prokinetic agent or laxative treatments 14 days before enrollment.
3. Receiving pediatric *Tuina* or probiotics 14 days before enrollment.
4. Children with Hirschsprung’s disease.
5. Children with neurological and psychiatric disorders (e.g., cerebral palsy, autism, spina bifida, anorexia nervosa).
6. Children with any organic or metabolic diseases of hematopoietic, endocrine, or immune systems.

### Drop out criteria

1. The children’s parents voluntarily asked to withdraw.
2. Patients have a sudden illness during the study.
3. Patients receive other pharmacological or non-pharmacological interventions during the study period.
4. Patients experience severe adverse reactions during the study.
5. The stool diary records are largely missing and incomplete.

### Randomization and blinding

Eligible FC children will be randomly assigned into two groups at a 1:1 ratio, based on the random number. There are two different types of interventions in this study. It is impossible to blind therapists and children’s parents or legal guardians. Nonetheless, outcome assessors and the data analyst will be blinded to the procedure and results of randomization, group allocation, and intervention.

### Sequence generation, allocation and the implementation

The randomization will be conducted by using a computerized random number generator, the Clinical Research Center of the Yunnan University of Chinese Medicine. There will be opaque envelopes with random numbers for random allocation. The envelopes will be managed by an independent member who is not involved in the treatment procedure or data analysis. The opaque envelope will not be opened until the participant is about to receive treatment, and then the participant will be grouped.

### Interventions

To ensure all manipulations follow the standard procedures. The therapists in this trial own an official medical license and hold a master’s degree in pediatric *Tuina*. During this period, participants are not allowed to use any other interventions, at least in principle. If participants take any other interventions in this period, they will be defined as non-adherent and will be dealt with drop out.

#### Pediatric *Tuina* group

Participants will receive pediatric *Tuina* treatment for 20 min each session. Once 2 days for four times. Treatment will be performed with the child lies in bed, with the placate by parents or legal guardians at the bedside. The standard pediatric *Tuina* includes 7 acupoints, which are determined by the literature review [27], and the pediatric *Tuina* professor with 30 years’ experience. Manipulation times is depended on the children’s age. Detailed information of acupoints, manipulation methods, and times is provided in Table 2. The location of acupoints and manipulations are shown in Additional file 2. The manipulations are light, fast, and gentle.

**Table 2:** Acupoints, manipulation methods and times

Age	Times							
	Large intestine meridian, reducing, pushing	Lung meridian, reducing, pushing	*Liufu*, reducing, pushing	*Sanguan*, reinforcing, pushing	Abdomen, clockwise, rubbing, and vibration	*Tianshu*, vertical, pressing, and vibration	*Qijiegu*, downward, pushing	*Guiwei*, clockwise, rubbing
≥ 1 year old and < 4 years old	300	200	200	20	300	300	300	200
≥ 4 years old and < 7 years old	500	400	400	40	500	500	500	400

#### Medilac-Vita group

According to the guideline of the Chinese Preventive Medicine Association (CPMA) in 2017, probiotics such as Medilac-Vita are recommended for the treatment of FC in children [28]. Participants assigned to this group will receive probiotics treatment. Medilac-Vita (combined bacillus subtilis and *enterococcus faecium* granules with multivitamins, live), which has been widely used for FC in children [29–32]. Participants will take 1 g (1 to 4 years old) or 2 g (4 to 7 years old) Medilac-Vita (Hanmi Pharm Co., Ltd., Beijing, China) dissolved in 100 ml water (40 °C), according to the drug instruction (Additional file 3). Twice a day, 7 consecutive days in total. After the treatment period, parents or guardians of the participants will be required to return any untaken drugs to avoid adverse consequences for follow-up.

### Outcomes measurement

A printed or electronic case report form (CRF) with the random number for each participant will be used to record all information. Three assessors who are blind to the group allocations will collect the basic information and assess the outcomes every week from the baseline to the end of follow-up. The first one will assess outcomes from the Second Affiliated Hospital of the Yunnan University of Chinese Medicine, Yunnan Province Hospital of TCM, and Kunming Children’s Hospital. The second one is for Zhejiang Provincial People’s Hospital. The third one is for Shandong Province Hospital of TCM.

#### Stool diary

Parents or legal guardians are required to keep a stool diary from baseline to intervention period and follow-up period. The stool diary contains 5 domains: weekly spontaneous bowel movements (SBMs), weekly complete spontaneous bowel movements (CSBMs), stool form, distress sensation, and glycerine enema rate.

#### Primary outcomes

The primary outcomes are weekly SBMs and/or CSBMs. Children older than 4 years old could answer whether defecation is complete. The assessors will check the stool diary and gather the data from baseline until the end of the follow-up.

#### Secondary outcomes

The secondary outcomes are effective rate, stool form, distress sensation, and glycerine enema rate. The effective rate is the number of participants who are effective (on average, at least one bowel movement in 3 days, stool softening, defecate unobstructed) divided by a total number of the group. Stool form will be measured according to the modified Bristol stool form scale (m-BSFS) (Table 3), which classifies the form of children’s feces into 5 types from separate hard lumps (type 1) to entirely watery (type 5) [33, 34]. Distress sensation measured in the range of 0–3 represents the intensity of distress during defecation, “0” means no cry or grimace, “1” means slight cry and grimace, “2” means medium cry and grimace, and “3” means hysterical cry and grimace. Glycerine enema rate is the times of using glycerine enema divided by the times of defecation, within a week.

**Table 3:** Modified Bristol stool form

**Type**	1	2	3	4	5
**Sample**					
**Character**	Separate hard lumps	Sausage-shaped but lumpy	Like sausage or snake, smooth and soft	Fluffy, ragged edges, mushy stool	Waterly, no solid pieces
**Score**	1	2	3	4	5

#### Safety assessment and dispose

The type, time of occurrence, severity, and duration of the adverse events will be recorded in detail from days 0 to 28. If emesis, severe diarrhea, dehydration, skin injury, fainting, infection, or other severe adverse events occur, the participants should be discontinued immediately from treatment and given appropriate treatment directly at the located hospital. And then, the detailed adverse event will be reported to the Ethics Committee.

### Adherence improvement

Parents will be offered free health counseling during the study. After the dropout or at the end of the full treatment, parents will be asked to hand over the remaining probiotics. Post the study, if the children still have constipation symptoms, they will be given three free pediatric *Tuina* sessions or two courses of probiotics.

### Sample size

A previous relevant study showed the effective rate of pediatric *Tuina* was 92.5% and Medilac-Vita was 72.5% [35]. Based on our own clinical experience, we consulted pediatric *Tuina* and physician experts. We anticipate an effective rate of 90% in the pediatric *Tuina* group and 70% in the Medilac-Vita group. Considering *α* = 0.05, 1-*β* = 0.90, *p*_1_ = 0.90, *p*_2_ = 0.70, *u*_0.05/2_ = 1.96, *u*_0.1_ = 1.282 [36], $n=1641.4\times {\left[\frac{\left(1.96+1.282\right)}{\sin \sqrt[-1]{0.90}-\sin \sqrt[-1]{0.70}}\right]}^2$ = 79.02 ≈ 79. With a drop-out of 10%, 88 FC children will be finally recruited in each group. Totally, 176 participants will be finally recruited in this study.

### Data collection

In this study, an independent data administrator will collect data every 3 months, from three assessors. The data of each participant includes basic information and outcomes. The basic information includes the participant’s gender, age, height, weight, history of pregnancy and delivery, past medical history, present condition, phone, and WeChat number of parents or legal guardians. Outcomes will be collected four times from baseline to the end of follow-up.

### Data monitoring

After the collection, the independent data administrator will check the CRFs to ensure data integrity and continuity. If in doubt, the stool diary and CRFs will be checked.

### Data management

The CRFs completed in 5 hospitals will be sent to the research office based in the School of Second Clinical Medicine, Yunnan University of Chinese Medicine. Two team members who are blind to the group allocations will perform double-data entry and summarize the data into an electronic dataset. Meanwhile, the data administrator will monitor and check for errors again. The data administrator and statistician will be blind to the group allocations and have the right to access the final dataset.

### Statistical analysis

The statistician will perform statistical analysis by using SPSS 19.0 statistics software (IBM Co., Armonk, NY, USA) and GraphPad 7.04 statistics software (GraphPad Software Co., San Diego, California, USA). Continuous variables are presented as the mean and standard deviation (SD). While the nonnormally distributed variables are presented as the median and interquartile range (IQR). Categorical variables are described as numbers and percentages (%).

Before the comparison, the normality and homogeneity of variance will be tested. Normality of continuous variables (SBMs, CSBMs, m-BSFS, distress sensation, and glycerine enema rate) will be determined by *Shapiro-Wilk* test, *Kolmogorov-Smirnov* test, *Skewness*, and *Kurtosis*. Homogeneity of variance of continuous variables will be determined by *F* test, *Brown-Forsythe*, and *Bartlett’s* test. Independent-Sample *t*-test or nonparametric test (*Mann-Whitney* test) will be used when compared between two groups. Paired *t*-test or nonparametric test (*Wilcoxon* test) will be performed to compare within a group. For the categorical variables (effective rate), the *χ*^2^ test will be performed to evaluate the significance of the difference. The statistical analysis will be a 2-sided test with a significance level of 0.05.

The missing data were will be dealt with expectation maximization, last observation carried forward method or listwise deletion. Subgroup analysis will be conducted according to the subject’s age (< 4 years and ≥ 4 years), which depends on the normality and homogeneity of variance of the data.

## Discussion

Pediatric *Tuina* is a Chinese massage therapy based on the special meridian-acupoint theory of TCM. Due to the characteristics of pediatric *Tuina*, all manipulations are performed on the body surface. Under some circumstances, cloaks and other covering may be used to blind parents or guardians [37]. However, most parents hope that child’s symptoms get a quick relief, despite FC is not life-threatening. In case of the efficacy (sham *Tuina*) cannot be guaranteed, it easily leads to doctor-patient disputes. Therefore, this study set probiotics with definite efficacy as the control.

Sample size calculation should be based on the results of relevant high-quality studies by principle. Limited by the methodological flaws of the existing studies, there were no objective indicators (e.g., SBM, CSBM, BSFS, m-BSFS) as outcomes. The effective rate was most widely used in pediatric *Tuina* for FC. Therefore, the sample size of this study set an effective rate as a reference. However, this study also set the defecation frequency, stool form, and distress sensation those directly related to constipation as the outcomes. Under the circumstance that lumpy stool and difficult defecation, glycerine enema is usually used to assist defecate. Anal injection of glycerine enema can lubricate the colon to promote the excretion of stool. Therefore, glycerine enema rate can indicate whether difficult defecation and whether the child can complete defecating on his own. Other evaluation indicators such as Cleveland Constipation Score (CCS), Patient Assessment of Constipation Symptom (PAC-SYM), and Patient Assessment of Constipation Quality of Life Questionnaire (PAC-QoL) are not suitable for children with FC, because children (especially toddlers) could not express their feelings directly and accurately [38–41].

**Fig. 2:**
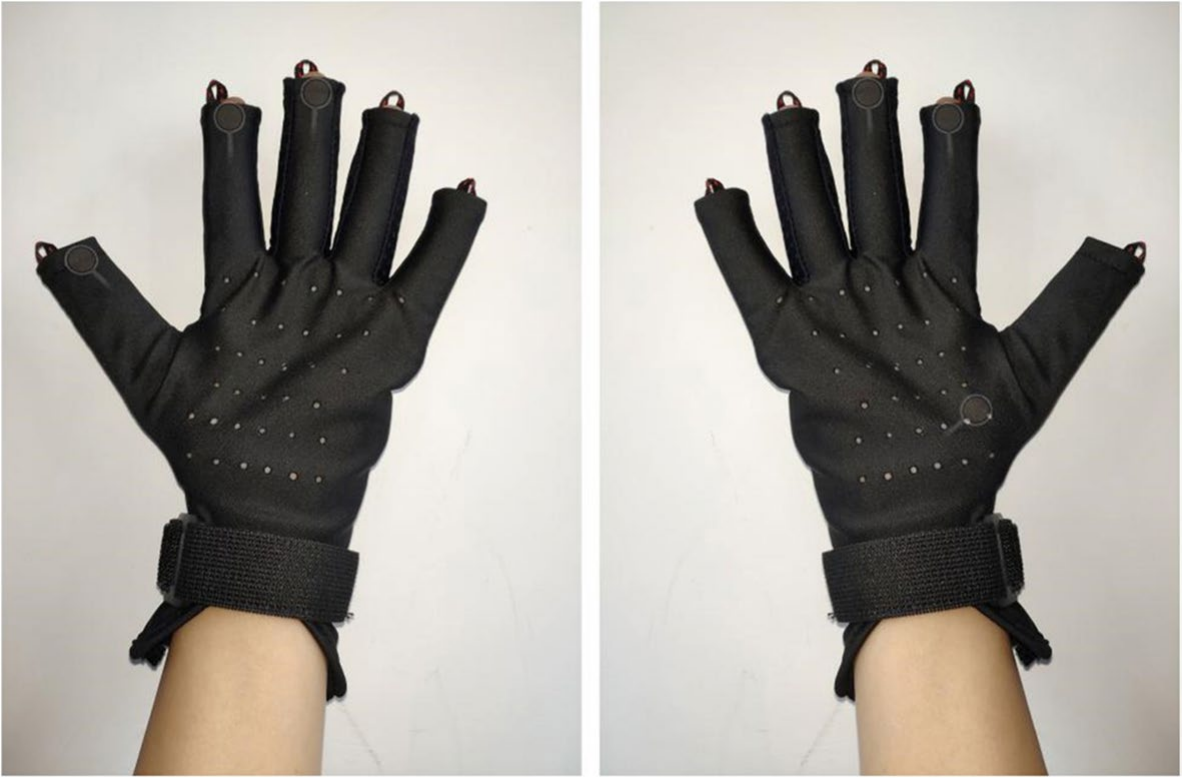
Electronic test glove

As a complementary and alternative therapy, standardization and quantification of massage manipulations have always been stumbling blocks which limit the scientific research and development of massage. The complex manipulations of massage determine that the robot can not completely replace the therapists. In China, pediatric *Tuina* is a medical treatment rather than a nursing skill. Although all manipulations will be performed by physicians with a master’s degree in pediatric *Tuina*, differences in manipulations may still exist between different physicians. In order to solve the differences, we will conduct unified training of pediatric *Tuina* before the study started. Electronic test glove which can measure pressure, amplitude, frequency, and angular velocity will be used to examine the therapist’s manipulation (Fig. 2 and Additional file 3).

In conclusion, this study may be the first high-quality RCT to certify the efficacy, safety, and advantage of pediatric *Tuina*. Since the probiotics’ curative effect is questioned in recent years [23], this study also investigates the differences in defecation frequency and stool form between pediatric *Tuina* and probiotics for children with FC. Once the objectives are achieved, it could provide strong evidence support for the enactment of clinical guidelines for FC. Pediatric *Tuina* may become an officially recognized treatment by the state, it may also be included in the category of medical insurance. The international influence and recognition of TCM pediatric *Tuina* will be greatly enhanced. It can also enhance the professionals’ confidence in the treatment of FC by pediatric *Tuina* After the study is completed, academic conferences and training courses may be performed to disseminate the pediatric *Tuina* for FC in children.

### Trial status

This trial was registered on 16 May 2021. The trial is currently in the stage of unified training of pediatric *Tuina.*

## Supplementary Information

**Additional file 1.** SPIRIT 2013 Checklist.

**Additional file 2 **Demonstration video of pediatric *Tuina*.

**Additional file 3.** Demonstration video of electronic test glove.

**Additional file 4.** Instruction of Medilac-Vita.

## Data Availability

No data were used to support this protocol. The outcomes data of the trial will be published within the final study manuscript and as the attachment for access. The outcomes data will also be uploaded to the Chinese Clinical Trial Registry.

## Abbreviations

CPMA: Chinese Preventive Medicine Association
CRF: Case report form
CSBMs: Complete spontaneous bowel movements
FC: Functional constipation
FGID: Functional gastrointestinal disorder
IQR: Interquartile range
m-BSFS: Modified Bristol stool form scale
RCTs: Randomized controlled trials
SBMs: Spontaneous bowel movements
SD: Standard deviation
SPIRIT: Standard Protocol Items Recommendations for Interventional Trials
TCM: Traditional Chinese medicine
