# Supplementary figures and images for: Pediatric Tuina for functional constipation in children: study protocol for a randomized controlled trail
Source: Trials. 2022 Sep 5;23:750. doi: 10.1186/s13063-022-06678-y (PMC9446667; doi:10.1186/s13063-022-06678-y)

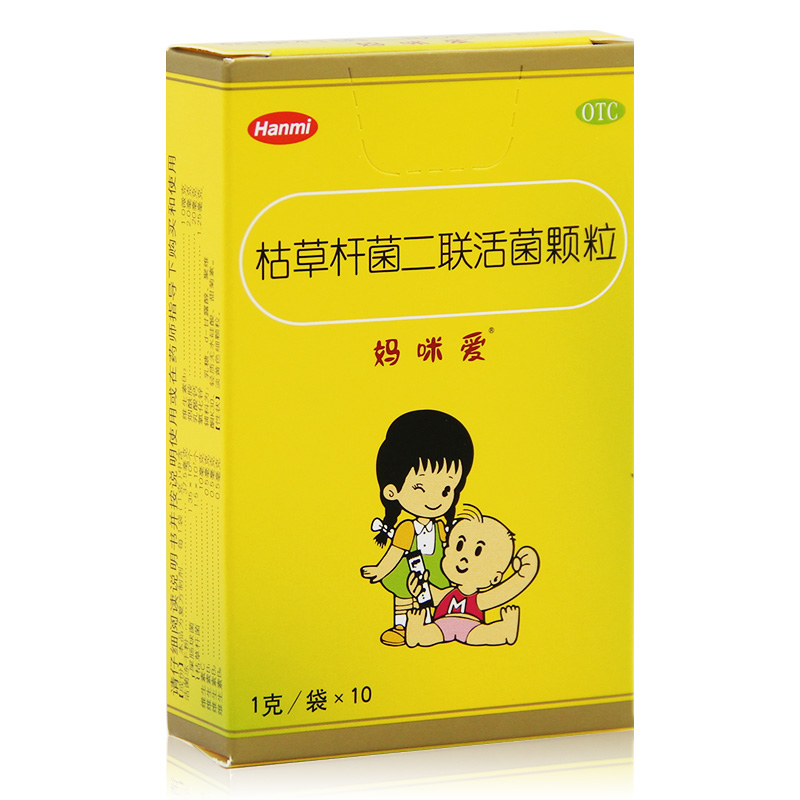

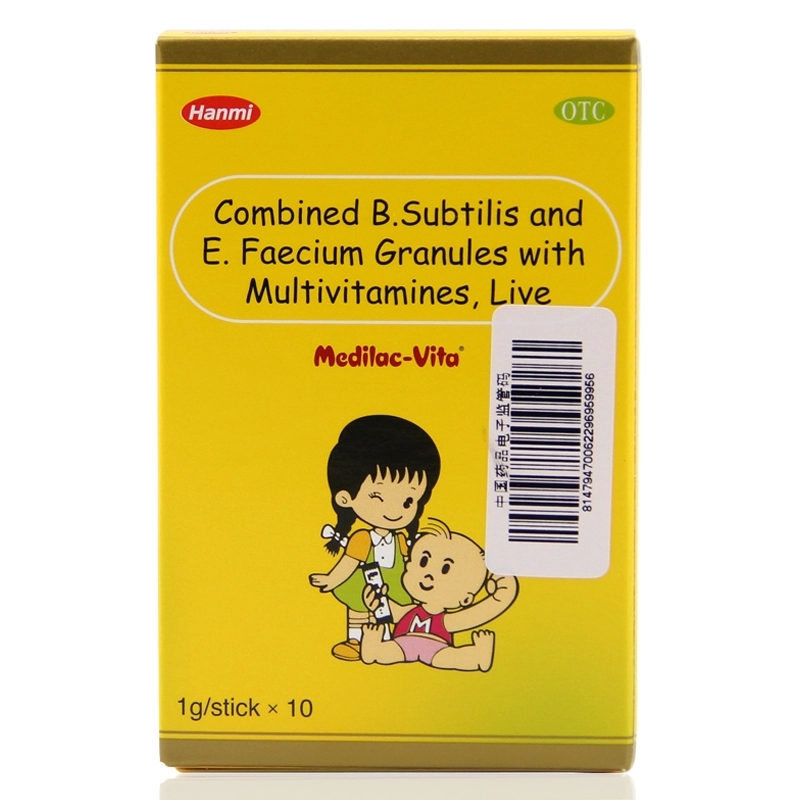

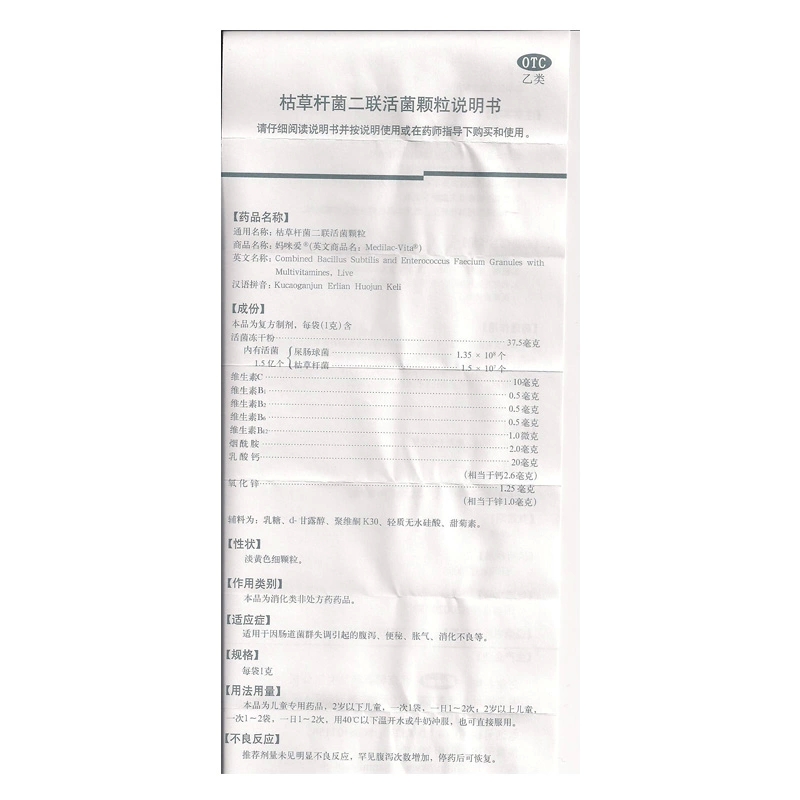

Supplement: Supplementary file 4 — Additional file 4. Instruction of Medilac-Vita. [file 13063_2022_6678_MOESM4_ESM.docx]
